# Optimization of Dairy Sludge for Growth of *Rhizobium* Cells

**DOI:** 10.1155/2013/845264

**Published:** 2013-09-09

**Authors:** Ashok Kumar Singh, Gauri Singh, Digvijay Gautam, Manjinder Kaur Bedi

**Affiliations:** Department of Microbiology, Dolphin (PG) Institute of Biomedical and Natural Sciences, Manduwala, Dehradun, Uttarakhand, India

## Abstract

In this study dairy sludge was evaluated as an alternative cultivation medium for *Rhizobium*. Growth of bacterial strains at different concentrations of Dairy sludge was monitored. Maximum growth of all strains was observed at 60% Dairy sludge concentration. At 60% optical density (OD) values are 0.804 for *Rhizobium trifolii* (MTCC905), 0.825 for *Rhizobium trifolii* (MTCC906), and 0.793 for *Rhizobium meliloti* (MTCC100). Growth pattern of strains was observed at 60% Dairy sludge along with different synthetic media (tryptone yeast, *Rhizobium* minimal medium and yeast extract mannitol). Growth in 60% Dairy sludge was found to be superior to standard media used for *Rhizobium*. Media were optimized using 60% dairy sludge along with different concentrations of yeast extract (1–7 g/L) and mannitol (7–13 g/L) in terms of optical density at different time intervals, that is, 24, 48 and 72 hours. Maximum growth was observed in 6 g/L of yeast extract and 12 g/L of mannitol at 48-hour incubation period in all strains. The important environmental parameters such as pH were optimized using 60% dairy sludge, 60% dairy sludge +6 g/L yeast extract, and 60% dairy sludge +12 g/L mannitol. The maximum growth of all strains was found at pH 7.0. The present study recommends the use of 60% dairy sludge as a suitable growth medum for inoculant production.

## 1. Introduction

The first step in the production of legume inoculants is massive growth of a selected Rhizobial strain in liquid medium [1]. As in case of many other industrial fermentations, the economy of such a process is largely governed by the price of media utilized. The suitability of culture media for large-scale production of *Rhizobium* depends upon the utilization of carbon source and multiplication rate of bacteria. For industrial production of rhizobial inoculants, it is important to identify inexpensive and easily available sources of nutrients for culture medium. Nutrient media such as yeast extract mannitol, tryptone yeast extract, and rhizobial minimal media are found to be very suitable for the growth of rhizobia. The standard medium includes mannitol, sucrose or glycerol as the carbon source, yeast extract as a source of nitrogen, growth factors, and mineral salts. The YMB medium (Yeast Mannitol Broth) has been mostly used for a laboratory-scale production [2–4]; however, its industrial use is limited due to high cost [5].

 In view of the growing demand of rhizobial inoculants it is important to search cheap and readily available substances against these expensive ingredients. Many investigators have looked for ways of producing biofertilizers using low-cost media. A variety of agricultural and industrial byproducts such as proteolyzed pea husks and water hyacinth [6], malt sprouts [7], deproteinized leave extracts [8], bagasse [9], waste water sludge [10], Jaggery solution [11], and sugar waste [12] have been used for commercial production of inoculants. All these materials contain growth factors, nitrogen and carbon for growth of various strains of *Rhizobium* equal to or better than the known growth in the available media.

In the present study, this aim was achieved for fast-growing rhizobia, using the dairy industry sludge as a suitable growth medium. *Rhizobium meliloti* (MTCC100), *Rhizobium trifolii* (MTCC 905), and *Rhizobium trifolii* (MTCC 906) strains obtained from Microbial Type Culture Collection (MTCC) from the Institute of Microbial Technology (IMTECH), Chandigarh, India were used as model strains.

The effluents are generated from milk processing through milk spillage, drippings, washing of cans, tankers bottles, utensil, equipments, and floors. The dairy industry generates an average of 2.5–3.0 liters of wastewater per liter of milk processed. Dairy sludge contains casein, lactose, fat, and valuable N, P, K, and organic matter. Dairy sludge has lower levels of heavy metals and other harmful components than waste water sludge and molasses used for cultivating *Rhizobium*.

## 2. Materials and Methods

### 2.1. Strains and Substrate Used

*Rhizobium meliloti *and *Rhizobium trifolii* strains were used. The dairy sludge was collected from Anchal Dairy which is situated in Dehradun on Sehestra Dhara Road. It is an effluent from dairy plant that is not treated and released as such. This dairy is well known in Dehradun for milk products like curd, cheese, and butter.

### 2.2. Starter Culture

50 mL of culture was prepared by inoculating *Rhizobium *strains into Yeast Extract Mannitol Broth (YEM). Starter culture was sterilized through autoclaving and incubated at 28 ± 2°C for 24 hrs.

### 2.3. Optimization of Dairy Sludge and Comparison with Different Lab Media

1% of starter culture was inoculated into different concentrations of Dairy sludge, that is, 2%, 4%, 6%, 8%, 10%, and 20% up to 100% and growth was monitored by recording optical density (OD) at regular time intervals. 

Effect of different media that is, 60% Dairy sludge, RMM, TY, and YEM on *Rhizobium* was monitored in terms of absorbance at 600 nm after every 24 hrs up to 72 hrs by inoculating the bacteria with these media.

### 2.4. Optimization of Dairy Sludge with Components of Standard Media

Yeast extract and mannitol are two main components of the standard media which are very helpful for the growth of *Rhizobium*. So the growth of bacteria was also observed in 60% sludge along with different concentrations of yeast extract (1 gm/L, 2 gm/L, and 3 gm/L up to7 gm/L) and similarly in 60% sludge along with different concentrations of mannitol (7 gm/L, 8 gm/L, and 9 gm/L up to 12 gm/L).

### 2.5. Effect of pH on Growth

*Rhizobium* strains were inoculated into 60% dairy sludge, 60% dairy sludge +6 g/L yeast extract and 60% dairy sludge +12 g/L mannitol as suitable media, maintained at different pH, that is, 6.0, 6.5, 7.0, 7.5, and 8.0, and growth was monitored in terms of absorbance at 600 nm after every 24 hrs up to 72 hrs. 

## 3. Results

### 3.1. Effects of Concentration of Dairy Sludge on Growth of *Rhizobium *Strains

**Figure 1:**
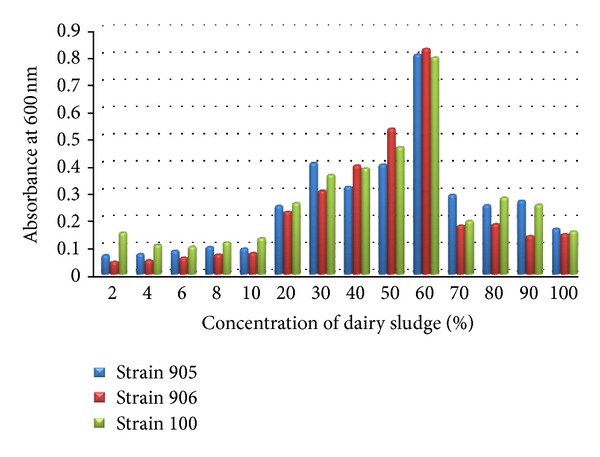
Growth of *Rhizobium* on different concentrations of dairy sludge.

Growth of *Rhizobium trifolii *(MTCC905),* Rhizobium trifolii *(MTCC906), and *Rhizobium meliloti *(MTCC100) at different concentrations of Dairy sludge (2%, 4%, 6%, 8%, 10%, 20%, 30%, 40%, 50%, 60%, 70%, 80%, 90%, and 100%) was monitored by recording optical density (OD) at 600 nm after 48 hrs incubation. There was a considerable decline in OD values after 60% Dairy sludge concentration. At 60% concentration OD values are 0.804 for *Rhizobium trifolii *(MTCC905), 0.825 for *Rhizobium trifolii *(MTCC906), and 0.793 for *Rhizobium meliloti *(MTCC100). However effect of Dairy sludge concentration (above 60%) was clearly visible on the growth of above three strains (Figure 1). Minimum growth for* Rhizobium trifolii *(MTCC905) and *Rhizobium trifolii *(MTCC906) was observed at 2.0% Dairy sludge concentration that is, 0.071 and 0.047, respectively, while minimum growth of *Rhizobium meliloti *(MTCC100) was observed at 10% Dairy sludge concentration, that is, 0.133.

### 3.2. Growth of *Rhizobium* Strainson Dairy Sludge Concentration and Synthetic Medium (YEM, RMM, and TY Media)

**Figure 2:**
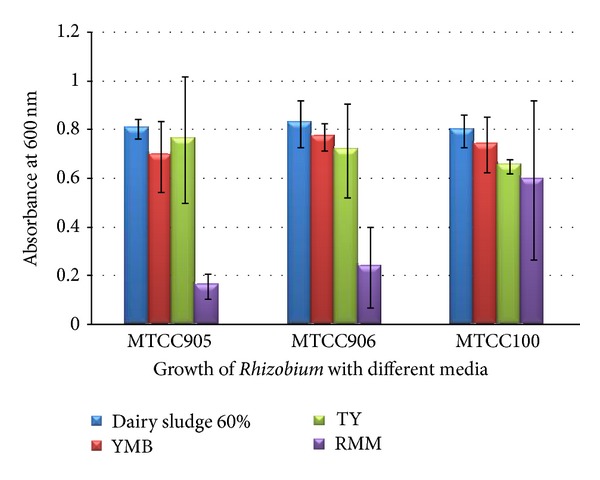
Comparison of growth of *Rhizobium* on different media with 60% dairy sludge.

 Growth of *Rhizobium *strains was observed at 60% Dairy sludgeconcentration along with YEM, RMM, and TY Media separately within the same conditions. Growth of *Rhizobium *strains was found maximum in 60% Dairy sludge concentration at 48 hrs, that is, 0.625 in *Rhizobium trifolii *(MTCC905), 0.804 in *Rhizobium trifolii *(MTCC906), and 0.793 in *Rhizobium meliloti *(MTCC100) and found minimum in RMM Medium in all strains used in the current study (Figure 2). 

### 3.3. Media Optimization Using 60% Dairy Sludge along with Different Concentrations of Yeast Extract and Mannitol

**Figure 3:**
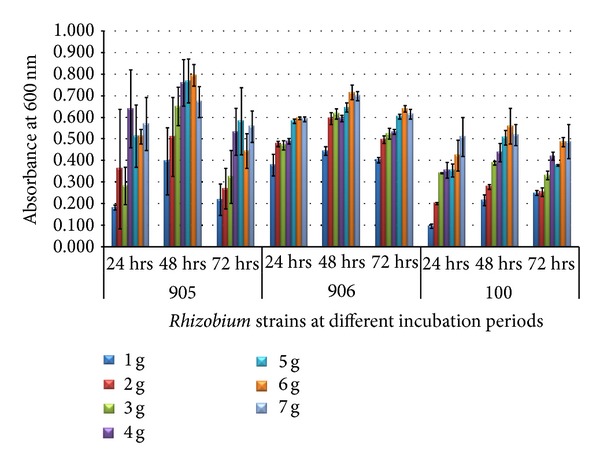
Growth of *Rhizobium* on 60% dairy sludge at various concentrations of yeast extract at different incubation periods.

**Figure 4:**
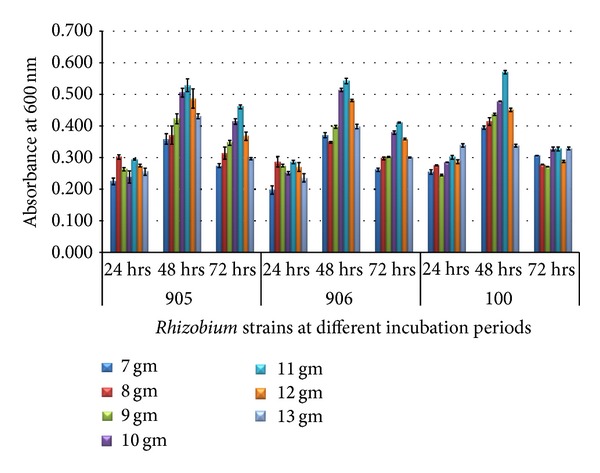
Growth of *Rhizobium* on 60% dairy sludge at various concentrations of mannitol at different incubation periods.

*Rhizobium* strains were exposed to various concentrations of yeast extract (1–7 g/L) and mannitol (7–13 g/L) in terms of optical density at different time intervals, that is, 24, 48, and 72 hours. Rhizobial cell viability was maintained throughout the 72-hour growth period. Maximum growth was observed in 6 g/L of yeast extract (Figure 3) and 12 g/L of mannitol (Figure 4) at 48-hour incubation period in all strains.

### 3.4. Combinations Effects of Dairy Sludge, Yeast Extract, and Mannitol at Different pH

**Figure 5:**
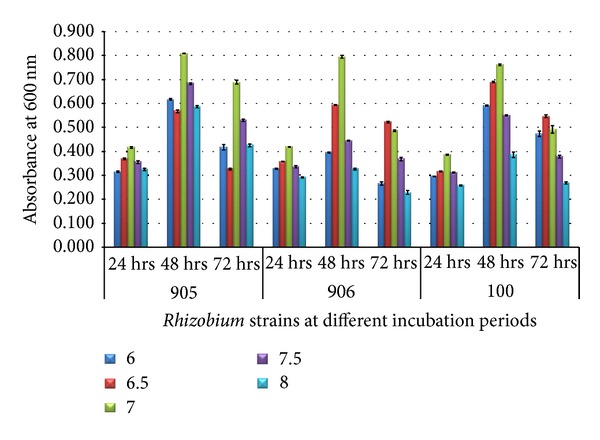
Growth of *Rhizobium* on 60% dairy sludge at various substrate at different incubation period.

At 28°C and 60% of dairy sludge, a considerable increase in OD values was observed reaching a maximum of 0.808 ± 0.001 in *Rhizobium trifolii *(MTCC905), 0.795 ± 0.006*in Rhizobium trifolii *(MTCC906), and 0.762 ± 0.004 in *Rhizobium meliloti *(MTCC100) with increasing pH up to 7.0 in 48 hrs (Figure 5). 

**Figure 6:**
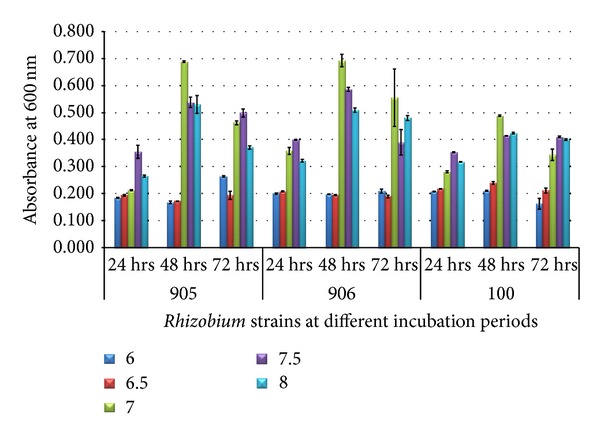
Growth of *Rhizobium* on 60% dairy sludge with yeast extract with different pH at different incubation periods.

At 28°C and 6.0 g/L yeast extract, growth of *Rhizobium *strains at different pH was recorded in terms of optical density (600 nm) at different time intervals (24, 48, and 72 hours) (Figure 6). There was a significant increase in the OD values reaching a maximum of 0.689 ± 0.003 in* Rhizobium trifolii *(MTCC905), 0.693 ± 0.023 in *Rhizobium trifolii *(MTCC906), and 0.488 ± 0.003 in *Rhizobium meliloti *(MTCC100) with the increase in pH that is up to 7.0 in 48 h. 

**Figure 7:**
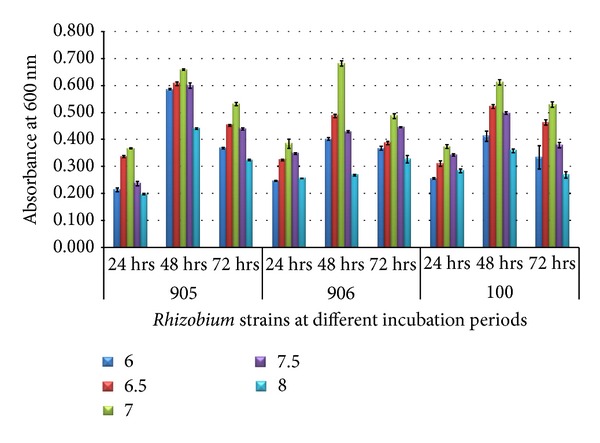
Growth of *Rhizobium* on 60% dairy sludge with mannitol at various pH with different incubation periods.

At 28°C and concentration of mannitol 12 g/L. growth of *Rhizobium trifolii *(MTCC905),* Rhizobium trifolii *(MTCC906), and *Rhizobium meliloti *(MTCC100) at different pH was recorded. There was an increase in the OD values reaching a maximum of 0.658 ± 0.003 in *Rhizobium trifolii *(MTCC905), 0.683 ± 0.010 in *Rhizobium trifolii *(MTCC906), and 0.612 ± 0.010 in *Rhizobium meliloti *(MTCC100) with the increase in pH that is up to 7.0 in 48 h. However, effect of pH (below and above 7.0) was clearly visible on the growth of strains (Figure 7).

## 4. Discussion

Dairy sludge obtained from Anchal Dairy which is situated in Dehradun (U.K.) is used as a growth media for fast growing rhizobia. Growth of *R. trifolii *and *R. meliloti *was observed at different concentrations of dairy sludge (2%, 4%, 6%, 8%, 10%, and 20% up to 100%) by recording optical density (OD) at 600 nm. Growth of *R. trifolii *and *R. meliloti* was maximum at 60% concentration. Similar work has been done using other substrates. Singh et al. [13] optimize different concentrations of sugar waste for fast-growing rhizobia and found maximum growth at 10% sugar waste. Ben Rebah et al. [10] demonstrated waste water sludge as a culture medium for rhizobia.

In the above study growth of all strains was increasing up to 60% dairy sludge, and above 60% concentration, fall in growth of *Rhizobium* was observed, which might be due to the fact that at higher concentrations of dairy sludge aeration conditions are improper in the medium. The aerobic condition is good for nitrogen-fixing bacteria (Hassen et al.) [14]. Therefore 60% concentration of dairy sludge was taken as optimum for further study.

The results of this study indicates that on medium containing only 60% dairy sludge, cells of fast-growing rhizobia (*R. meliloti* and *R. trifolii) *grow rapidly and growth was superior to that of the control (standard media). These results demonstrate that 60% dairy sludge can be satisfactorily used as a growth substrate of rhizobia. Annapurna et al. [15] used 1% aqueous solutions of commercial quality molasses, malt extract, jaggery, peptone, and yeast extract as the sole source of nutrients for the two species *of Rhizobium,* that is, *R. trifolii* (RC 1–4), a fast grower, and *R. Japonicum* (SB-16), a slow grower. YEM Broth was used as standard medium for comparison; among different media used jaggery solution supported maximum growth (10.17 for *R. trifolii* and 9.98 for *R. Japonicum) *followed by molasses (9.65 for *R. trifolii *and 9.22 for* R. Japonicum) *though it was not comparable to that of YEM Broth which supported 10.31 for* R. trifolii* and 10.13 for *R. Japonicum; *therefore, according to this finding jaggery solution and molasses can be used as an alternate of YEM Broth in commercial preparation of inoculants. This will considerably reduce the production cost. Similar observation has been reported by Daniel et al. (2009) [16]. The composition of the alternative culture media included glycerol, molasses, glutamate, yeast extract and salts for *Rhizobium *was optimized and no significant difference was observed in the alternate culture media, the traditional one (Yeast extract mannitol agar medium). Estrella et al. [17] found that cheese whey-based growth medium efficiently protects *Rhizobium loti* cells during freezing and can maintain their growth in a manner similar to that of traditional mannitol-based medium (YEM).

The concept of media optimization was to establish a medium which shows the optimum conditions for the growth of the organism at cheap cost as compared to normal media. *Rhizobium *strains were able to utilize glucose and sucrose more efficiently than normal YEM medium [18]. Yeast extract and mannitol are two main components of the standard media for the growth of *Rhizobium; *therefore, 60% sludge along with different concentrations of yeast extract (1 gm/L, 2 gm/L, and 3 gm/L up to 7 gm/L) and 60% sludge with different concentrations of mannitol (7 gm/L, 8 gm/L, and 9 gm/L up to 13 gm/L) were further optimized. Growth in both conditions was studied at different time intervals that is, 24, 48, and 72 hours. Our results demonstrated that maximum growth was observed in 6 g/L of yeast extract and 12 g/L of mannitol at 48-hour incubation period in all strains. 

The effects of additional sources of nutrients on the growth of *S. meliloti *were studied in secondary sludge from the Quebec City Wastewater Plant (Ben Rebah et al.) [19]. Sludge supplemented with different concentrations of yeast extract and glycerol increased the maximum cell counts. The highest increase was approximately sixfold and was obtained with the addition of 4 g/L yeast extract and 7.5 g/L glycerol.

pH is an important parameter for the growth of organisms, slight variation in pH of medium might have enormous effects on the growth of organisms, and keeping this in mind, growth of fast growing strains was monitored at different pH (6.0, 6.5, 7.0, 7.5, and 8.0) at different time intervals, that is, 24, 48, and 72 hours. Conditions used were 60% dairy sludge, 60% dairy sludge +6 g/L yeast extract, and 60% dairy sludge +12 g/L mannitol.

The result of the effects of different pH values on the growth of rhizobia shows that these strains grow well at pH 7.0 on observing OD at 48 hrs incubation period. This is in accordance with Mensah et al. [20]; they recorded maximum absorbance for broth experiment as well as exhibited heavy growth as measured by population count at pH 7.0 for *Rhizobium* species. Ali et al. [21] also observed that there was a considerable increase in OD values with increasing pH up to 7.0. Rodrigues et al. [22] reported that pH 7.0 is the most optimum pH for the growth of root modulating bacteria. The above results indicate that optimum growth occurred at pH 7.0 and incubation period of 48 Hrs.

## 5. Conclusion

 Our study demonstrated for the first time that sludge generated by dairy industry sustains growth of fast-growing rhizobia higher than standard medium and different components used in YEM medium. The present study concludes that 60% dairy sludge is a suitable growth medium. Production cost of biofertilizers will be reduced by reusing the dairy industry sludge as a substrate.
